# External validation of Pentafecta in patients undergoing laparoscopic radical cystectomy: results from a high-volume center

**DOI:** 10.1186/s12894-022-00987-9

**Published:** 2022-03-21

**Authors:** Kai Li, Xiao Yang, Juntao Zhuang, Lingkai Cai, Jie Han, Hao Yu, Zijian Zhou, Jianchen Lv, Dexiang Feng, Baorui Yuan, Qikai Wu, Pengchao Li, Qiang Cao, Qiang Lu

**Affiliations:** Department of Urology, The First Affiliated Hospital of Nanjing Medical University, Nanjing, 210029 People’s Republic of China

**Keywords:** Bladder cancer, Pentafecta, Radical cystectomy, Pelvic lymph node dissection

## Abstract

**Background:**

To investigate whether Pentafecta is suitable for bladder cancer patients receiving laparoscopic radical cystectomy (LRC).

**Methods:**

From November 2013 to December 2020, muscle invasive Bladder Cancer (MIBC) and non-muscle invasive Bladder Cancer (NMIBC) patients who received LRC and urinary diversion were retrospectively analyzed. Pentafecta was defined as meeting five criteria: negative soft margin, ≥ 16 lymph nodes (LNs) removed, major complications free, urinary diversion related sequelae free and clinical recurrence free within 1 year. Analyze the achievement of five criteria and compare the overall survival (OS) of Pentafecta group with non-attainment group. Multivariable Cox’s regression was performed to evaluate the impact of Pentafecta on OS. Multivariable logistic regression was performed to explore the effect of surgical experience on Pentafecta attainment.

**Results:**

A total of 340 patients were included, negative soft margin, ≥ 16 lymph nodes (LNs) removed, major complications free, urinary diversion related sequelae free and clinical recurrence free within 1 year were observed in 95.3%, 30.3%, 83.8%, 75.0% and 85.6% of patients, respectively. Pentafecta group had a significantly longer OS than the non-attainment group (*P* = 0.027). The group with 10–15 LNs removed and meeting the other four criteria had a similar OS to group with ≥ 16 LNs removed (Pentafecta group) (5-year OS: 67.3% vs 72.7%, *P* = 0.861). Pentafecta (HR = 0.33, *P* = 0.011), positive lymph nodes (HR = 2.08, *P* = 0.028) and MIBC (HR = 3.70, *P* < 0.001) were all significant predictors of OS in multivariable Cox’s regression. Surgical experience (OR = 1.05, *P* < 0.001), conduit (OR = 2.09, *P* = 0.047) and neobladder (OR = 2.47, *P* = 0.048) were all independent predictors of Pentafecta attainment in multivariable logistic regression.

**Conclusions:**

Pentafecta is suitable for bladder cancer patients receiving LRC and has the potential to be a valuable tool for evaluating the quality of LRC. Based on Pentafecta analysis, removing 10 LNs instead of 16 LNs as the one of the five criteria may be more appropriate for bladder cancer patients.

**Supplementary Information:**

The online version contains supplementary material available at 10.1186/s12894-022-00987-9.

## Background

Bladder Cancer is the eleventh most common cancer globally [1]. Bladder cancers included NMIBC and MIBC. The 5-year OS rate for patients with MIBC is roughly 60% to 70% [2], while the 5-year survival rate of MIBC patients with distant metastasis is approximately 15% [3]. RC is the gold standard for the treatment of MIBC and high-risk NMIBC [1, 4].

However, RC is a demanding operation and that may result in many serious complications. The rate of postoperative complications following RC has been reported to range from 30 to 70% [5–9]. According to a recent prospective randomized trial, the incidence of postoperative complications was close to 70% regardless of whether open or robotic cystectomy was used [10]. According to the PURE-01 Trial, the rate of serious complications (Clavien–Dindo III–V) was 34% [11]. Additionally, the quality of surgery had a substantial effect on the oncological outcomes and overall survival of bladder cancer patients [12]. Although certain molecular markers have been investigated, early predictors for prognosis following RC remain lacking. Urologists have introduced some new treatment strategies, including laparoscopic or robot-assisted laparoscopic procedure and urinary diversion, necessitating the development of a systematic evaluation system for perioperative morbidity and oncology outcomes. Aziz et al. [13] initially introduced a concept of Pentafecta, and Cacciamani et al. [12] generated an updated version about RC that we used in this article.

The quantity of LNs is a critical metric for the Pentafecta. The number of LNs removed depends on the extent of pelvic lymph node dissection (PLND). PLND templates included limited, standard, extended and super-extended templates [14]. During RC for bladder cancer, PLND was superior than no PLND [1]. However, the threshold of PLND is controversial. In the definition of Pentafecta, a PLND threshold of 16 LNs was used. Herr et al. [15] reported, however, that at least 10 to 14 LNs should be retrieved in RC.

We examined the Pentafecta outcomes in the patients who received LRC with urinary diversion. By comparing the results of other studies, it was determined which criteria are more appropriate for bladder cancer patients.

## Methods

### Study population

From November 2013 to December 2020, MIBC and NMIBC patients who received LRC and urinary diversion at our center were retrospectively analyzed. The patients were followed for a minimum of 12 months.

### Pentafecta analysis

Patients who met all five criteria, including negative soft margin, the removal of ≥ 16 LNs, the absence of major complications (Clavien-Dindo grade III-V), the absence of urinary diversion related sequelae and clinical recurrence free within 1 year, were considered as having achieved the Pentafecta [12].

### Subgroup analysis

To explore whether the removal of 10 LNs could be the PLND threshold for Pentafecta, we compared patients with 10 to 15 LNs were removed who met the other four Pentafecta criteria to those with ≥ 16 LNs removed group (Pentafecta group) in terms of OS. Multivariable Cox’s regression was performed to evaluate the impact of Pentafecta (10 LNs as the threshold of Pentafecta) on OS. Then we conducted a subgroup analysis on between PLND in NMIBC and MIBC.

### Statistical analysis

All data were statistically analyzed by SPSS 26.0. Measurement data were expressed as mean and standard deviation, categorical data were expressed as number and percentage. All tests were two-tailed tests, *P* < 0.05 was considered statistically significant. Univariate analysis was performed using Kruskal–Wallis, chi-squared and Fisher’s exact tests to compare measurement and categorical data, as appropriate. Overall survival was analyzed by Kaplan–Meier analyses with the log-rank test. Multivariable Cox’s regression was performed to evaluate the impact of Pentafecta on OS. Surgical experience was coded as the number of prior LRC performed by each surgeon at the time of each patient’s surgery [16]. Multivariable logistic regression was performed to explore the effect of surgical experience on Pentafecta attainment. Multivariable binary logistic regression analysis was used to assess the factors leading to no PLND.

## Results

### Baseline characteristics

Total 340 patients were enrolled. 340 patients underwent LRC. The 340 patients received follow up at least 12 months. Median follow-up was 23.0 months, with an interquartile range (IQR) 14.0–38.5 months.

According to Table 1, the average age of the patients was 66.41 yr, of which 84.7% were male. 38.5% patients (131/340) had a smoking history, and 11.2% patients (38/340) received neoadjuvant chemotherapy (NAC). Pentafecta attained group was younger than Pentafecta not attained group (62.64 ± 10.36 vs. 67.07 ± 11.31, *P* = 0.01). In terms of urinary diversion, 173 (50.9%) received a cutaneous ureterostomy, 107 (31.5%) received ileum conduit and 60 (17.6%) received ileum orthotopic neobladder. Among the 340 included patients, 190 (55.9%) had ≥ pathological T2 and 28 (8.2%) had pathologically positive LN. In our center, the average number of LNs dissected per patients was 10.46 (Additional file 1).

**Table 1:** The clinicopathological characteristics among BCa patients

Characteristics	Total	Pentafecta attained	Pentafecta not attained	P value
Patients (n, %)	340 (100)	50 (14.7)	290 (85.4)	
Mean age, years ± SD	66.41 ± 11.27	62.64 ± 10.36	67.07 ± 11.31	0.01
Gender (n, %)				0.317
Male	288 (84.7)	40 (80.0)	248 (85.5)	
Female	52 (15.3)	10 (20.0)	42 (14.5)	
Smoking history (yes, %)	131 (38.5)	14 (28.0)	117 (40.3)	0.136
Neoadjuvant chemotherapy (n, %)	38 (11.2)	8 (16.0)	30 (10.3)	0.241
Type of urinary diversion (n, %)				0.014
Cutaneous ureterostomy	173 (50.9)	16 (32.0)	157 (54.1)	
Conduit	107 (31.5)	21 (42.0)	86 (29.7)	
Orthotopic neobladder	60 (17.6)	13 (26.0)	47 (16.2)	
Pathological T-stage (%)				0.488
pT0	29 (8.5)	1 (2.0)	28 (9.7)	
pTa	25 (7.4)	3 (6.0)	22 (7.6)	
pT1	96 (28.2)	17 (34.0)	79 (27.2)	
pT2	83 (24.4)	12 (24.0)	71 (24.5)	
pT3	65 (19.1)	9 (18.0)	56 (19.3)	
pT4	42 (12.4)	8 (16.0)	34 (11.7)	
Pathological N-stage (%)				0.408
N0	312 (91.8)	44 (88.0)	268 (92.4)	
N1	15 (4.4)	4 (8.0)	11 (3.8)	
N2	9 (2.6)	2 (4.0)	7 (2.4)	
N3	4 (1.2)	0 (0)	4 (1.4)	
No. lymph node removed (mean, ± SD)	10.46 ± 8.60	20.34 ± 4.23	8.76 ± 8.0	< 0.001

BCa = bladder cancer; SD = standard deviation

### Survival outcomes of Pentafecta

**Fig. 1:**
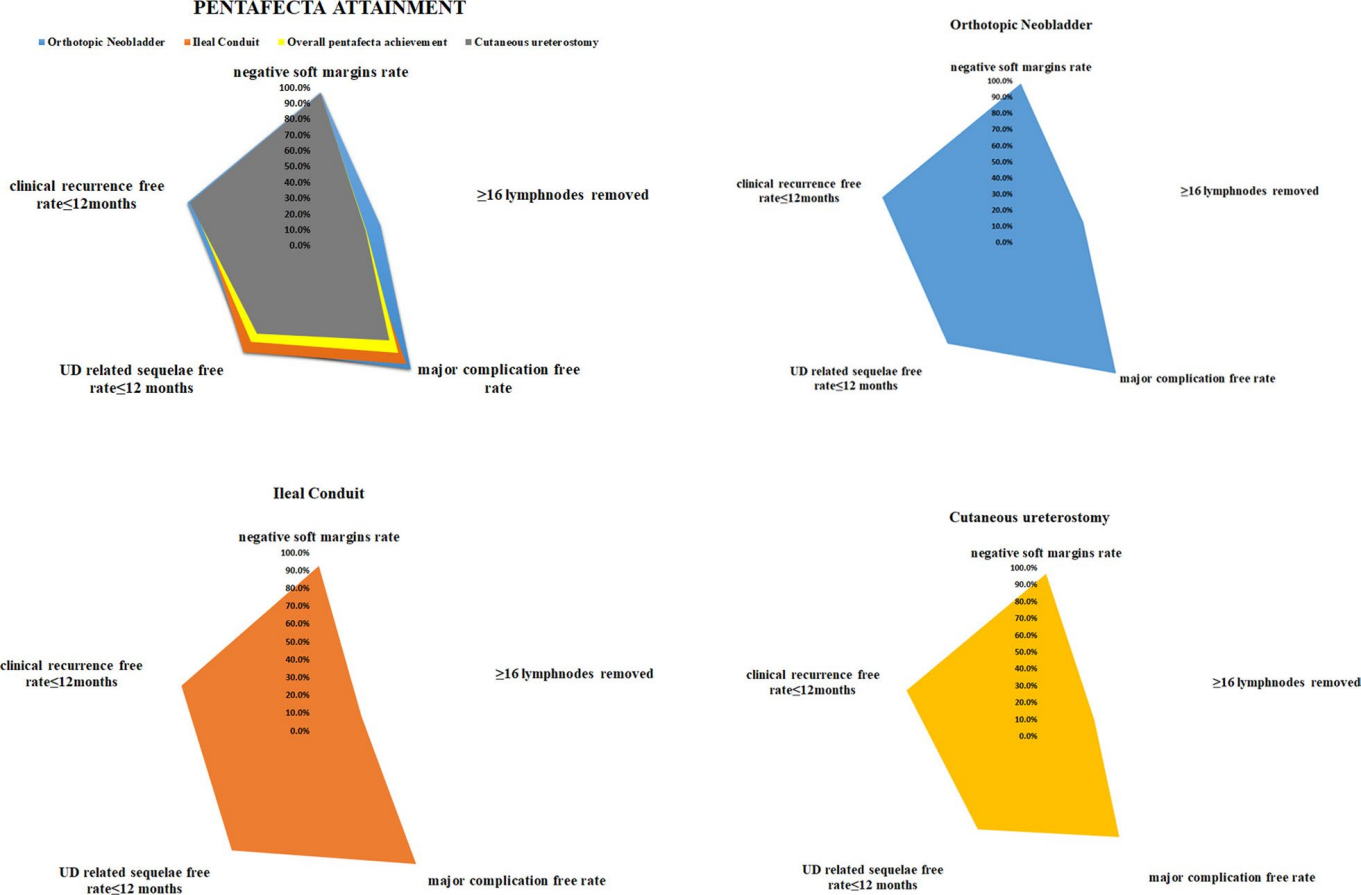
Achievement of Pentafecta

**Fig. 2:**
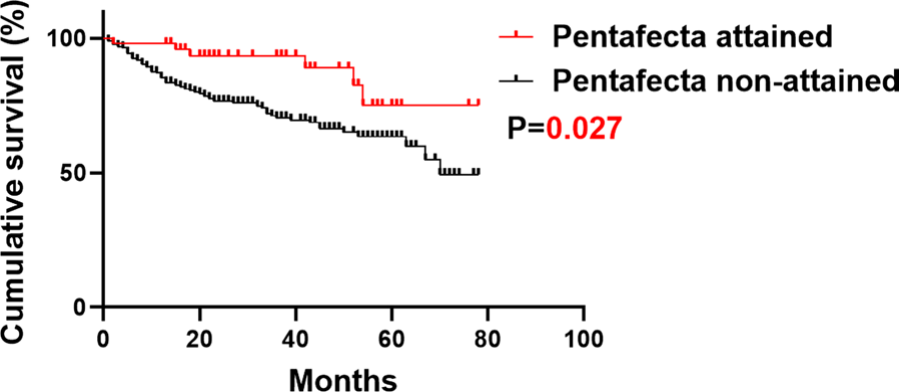
Overall survival (OS) between Pentafecta attained and non-attained group

Among the 340 included patients, 50 (14.7%) attained Pentafecta while 290 (85.3%) did not. Over the follow-up, each criterion of Pentafecta was observed in 95.3%, 30.3%, 83.8%, 75.0% and 85.6% of patients, respectively (Fig. 1). The median follow-up time was 23.0 months, and 88 (25.9%) of all patients died. The 5-year OS rate was 59.5 percent among 340 patients. Patients who achieved Pentafecta had a much better prognosis than those who did not (5-year OS: 72.7% vs. 63.8% *P* = 0.027) (Fig. 2). To rule out the effect of NAC on the prognosis, we also analyzed the impact of Pentafecta on survival in patients receiving NAC or not. In NAC patients, the Pentafecta attained group had similar OS compared with the Pentafecta not attained group (5-year OS: 100.0% vs. 86.7% *P* = 0.289) (Additional file 2: Figure S1A). In non-NAC patients, the Pentafecta attained group had significantly higher 5-year OS than the Pentafecta not attained group (5-year OS: 70.6% vs. 61.8%, *P* = 0.034) (Additional file 2: Figure S1B). At multivariable Cox’s regression, Pentafecta (HR = 0.33, *P* = 0.011), positive lymph nodes (HR = 2.08, *P* = 0.028) and MIBC (HR = 3.70, *P* < 0.001) were significant predictors of OS (Table 2).

**Table 2:** Multivariable Cox’s proportional hazard regression model to predict overall survival

Variable	HR (95% CI)	P value
Sex (male vs. female)	0.81 (0.44–1.50)	0.510
Age (≤ 65 vs. > 65)	0.68 (0.42–1.10)	0.113
Diversion type (conduit vs. cutaneous ureterostomy)	1.52 (0.96–2.41)	0.072
Diversion type (neobladder vs. cutaneous ureterostomy)	0.59 (0.26–1.36)	0.218
Pathological T-stage (MIBC vs. NMIBC)	3.70 (2.19–6.26)	< 0.001
Pathological N-stage (N + vs. N−)	2.08 (1.08–4.00)	0.028
Smoking history (yes vs. no)	1.23 (0.78–1.95)	0.381
Neoadjuvant chemotherapy (yes vs. no)	0.56 (0.20–1.57)	0.271
Pentafecta (yes vs. no)	0.33 (0.14–0.78)	0.011

### Subgroup analysis

**Fig. 3:**
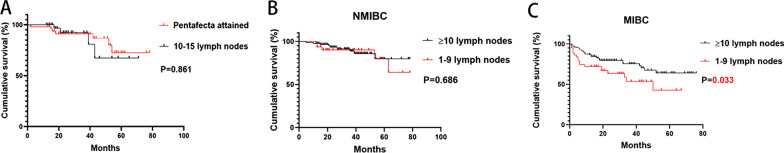
Subgroup survival analysis on the number of removed LNs. **A** Compare the group with 10–15 LNs removed and meeting the remaining four Pentafecta criteria to group with ≥ 16 LNs removed (Pentafecta group) in OS. **B** In NMIBC patients, compare the group with ≥ 10 LNs removed to group with < 10 LNs removed in OS. **C** In MIBC patients, compare the group with ≥ 10 LNs removed to group with < 10 LNs removed in OS

The group with 10–15 LNs removed and meeting the other four Pentafecta criteria had a similar OS to the group with ≥ 16 LNs (Pentafecta group) (5-year OS: 67.3% vs. 72.7%, *P* = 0.861) (Fig. 3A). At multivariable Cox’s regression, Pentafecta (10 LNs) (HR = 0.31, *P* = 0.029), positive lymph nodes (HR = 2.86, *P* = 0.008) and MIBC (HR = 4.96, *P* < 0.001) were significant predictors of OS (Table 3).

**Table 3:** Multivariable Cox’s proportional hazard regression model to predict overall survival in the subgroup (10 LNs as Pentafecta threshold)

Variable	HR (95% CI)	P value
Sex (male vs. female)	0.72 (0.38–1.38)	0.325
Age (≤ 65 vs. > 65)	1.07 (0.63–1.81)	0.817
Diversion type (conduit vs. cutaneous ureterostomy)	1.31 (0.80–2.16)	0.289
Diversion type (neobladder vs. cutaneous ureterostomy)	0.79 (0.36–1.84)	0.476
Pathological T-stage (MIBC vs. NMIBC)	4.96 (2.61–9.40)	< 0.001
Pathological N-stage (N + vs. N−)	2.86 (1.31–6.22)	0.008
Smoking history (yes vs. no)	0.92 (0.55–1.55)	0.756
Neoadjuvant chemotherapy (yes vs. no)	0.92 (0.32–2.61)	0.870
Pentafecta (10 LNs) (yes vs. no)	0.31 (0.11–0.89)	0.029

In terms of pathological stage, individuals with ≥ 10 LNs had a similar OS to those with < 10 LNs removed in NMIBC patients (5-year OS: 79.7% vs. 64.1%, *P* = 0.686) (Fig. 3B). However, the group with ≥ 10 LNs removed had a much longer OS than the group with < 10 LNs removed in MIBC patients (5-year OS: 63.9% vs. 53.7%, *P* = 0.033) (Fig. 3C).

**Fig. 4:**
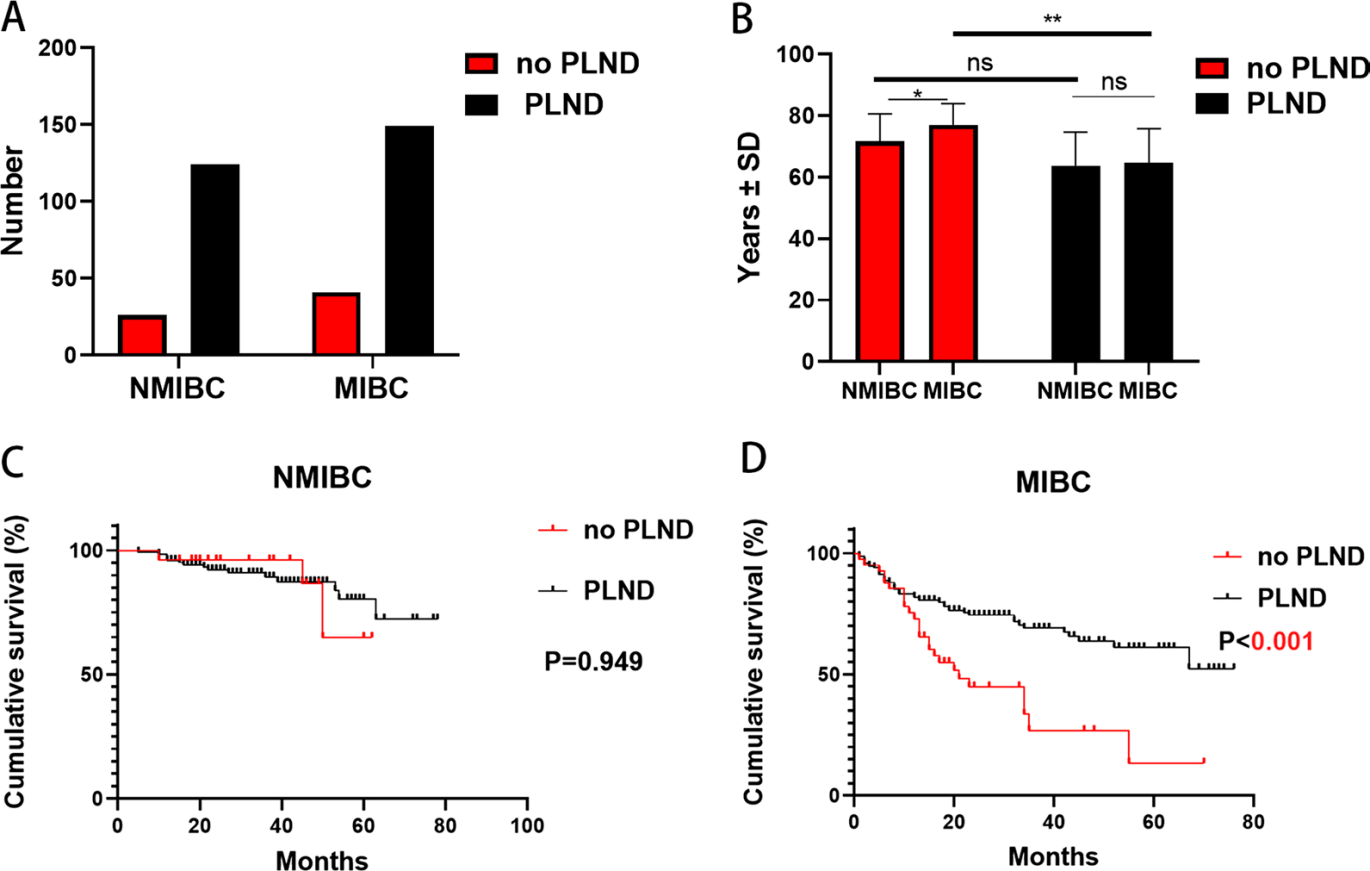
Overview of PLND. **A** The status of PLND both in NMIBC and MIBC. **B** In both NMIBC and MIBC, compare the age of patients in the PLND and no PLND group. **C** In NMIBC patients, compare the PLND group with no PLND group in OS. **D** In MIBC patients, compare the PLND group with no PLND group in OS

In NMIBC, 124 (82.7%) patients received PLND, while 26 (17.3%) patients didn't. In MIBC, 149 (78.4%) patients received PLND, while 41 (21.6%) patients didn't (Fig. 4A). In both NMIBC and MIBC, the average age of the patients with no PLND was higher than that of patients with PLND. Additionally, the average age of MIBC patients receiving PLND was higher than that of NMIBC patients (Fig. 4B). In NMIBC, the PLND group had a similar OS with no PLND group (5-year OS: 80.3% vs. 64.9%, *P* = 0.949) (Fig. 4C). In MIBC, the PLND group had a significantly longer OS than no PLND group (5-year OS: 60.9% vs. 26.9%, *P* < 0.001) (Fig. 4D). Another multivariable logistic regression analysis revealed that PLND execution was associated with younger age (≤ 65 years) (OR 3.33, *P* < 0.001) and NAC (OR 8.36, *P* = 0.041) (Table 4).

**Table 4:** Multivariable binary logistic regression analysis of the factors leading to PLND

Variable	OR (95% CI)	P value
Sex (male vs. female)	1.29 (0.58–2.87)	0.533
Age (≤ 65 vs. > 65)	3.33 (1.68–6.59)	0.001
Diversion type (conduit vs. cutaneous ureterostomy)	1.40 (0.74–2.65)	0.30
Diversion type (neobladder vs. cutaneous ureterostomy)	2.53 (0.81–9.10)	0.093
Pathological T-stage (MIBC vs. NMIBC)	0.73 (0.40–1.31)	0.286
Smoking history (yes vs. no)	1.36 (0.72–2.59)	0.345
Neoadjuvant chemotherapy (yes vs. no)	8.36 (1.09–64.12)	0.041

### Predictors of Pentafecta attainment

At multivariable logistic regression, conduit (OR = 2.09, *P* = 0.047), neobladder (OR = 2.47, *P* = 0.048) and surgical experience (OR = 1.05, *P* < 0.001) were independent predictors of Pentafecta attainment (Table 5).

**Table 5:** Multivariable logistic regression predictive model of Pentafecta achievement

Variable	OR (95% CI)	P value
Sex (male vs. female)	0.72 (0.31–1.67)	0.445
Age (≤ 65 vs. > 65)	1.51 (0.79–2.91)	0.214
Diversion type (conduit vs. cutaneous ureterostomy)	2.09 (1.01–4.31)	0.047
Diversion type (neobladder vs. cutaneous ureterostomy)	2.47 (1.01–6.05)	0.048
Pathological T-stage (MIBC vs. NMIBC)	1.10 (0.58–2.07)	0.780
Pathological N-stage (N + vs. N−)	1.68 (0.58–4.85)	0.339
Smoking history (yes vs. no)	1.36 (0.72–2.59)	0.345
Neoadjuvant chemotherapy (yes vs. no)	1.48 (0.60–3.67)	0.397
Surgical experience (continuous)	1.05 (1.03–1.07)	< 0.001

## Discussion

RC with Urinary Diversion is a complicated operation, where the technical quality of operation has a substantial impact on perioperative morbidity and oncological outcomes. Pentafecta can be used to evaluate the quality of surgery and predict survival prognosis, covering oncological outcomes, short and long-term complications.

In our study, a total of 340 patients were included, negative soft margin, ≥ 16 LNs removed, major complications free, urinary diversion related sequelae free and clinical recurrence free within 1 year were observed in 95.3%, 30.3%, 83.8%, 75.0% and 85.6% of patients, respectively. And 14.7% of patients achieved the Pentafecta.

Cacciamani et al. [12] and Piazza et al. [17] reported a 53.3% and 52.2% Pentafecta rate, respectively. Two multicenter studies by Oh JJ et al. [18] and Baron et al. [19] reported the Pentafecta rate of 28.5% and 39.4%, respectively. Oh JJ et al. and Baron et al. reported a lower Pentafecta rate than Cacciamani et al. and Piazza P et al. which could be attributed to their relatively low rate of ≥ 16 LNs removed. Their findings could be less biased as a result of their multicenter research. In comparison to previous research, our center had a relatively reduced rate of Pentafecta achievement. However, RARC and LRC had comparable complications, pathological and oncological outcomes, according to a meta-analysis [20]. We further explore the factors that contribute to our relatively low Pentafecta rate.

The primary causes for our center’s reduced Pentafecta rate were a relatively low urinary diversion related sequelae free rate (75.0%) and low rate of ≥ 16 LNs removed (30.3%). In our study, 50.9% patients received cutaneous ureterostomy, whereas previous Pentafecta studies involved only patients who received ileal conduit and neobladder [12, 17–19]. The cutaneous ureterostomy might improve surgical tolerance in the elderly patients, which cloud result in a reduced rate of perioperative and postoperative complications [21]. However, the remarkable long-term complication related to cutaneous ureterostomy was ureteral strictures, which occurred at a higher rate than those associated ileal conduit and neobladder [22], contributing to the high risk of urine diversion-related sequelae.

Additionally, we examined the status of LNs removed in our center to explore the effect on Pentafecta attainment, and tried to determine the optimal amount of LNs removed.

The number of LNs was determined by the range of PLND. PLND included templates that were limited, standard, extended and super-extended [14]. PLND was superior to no PLND during RC of bladder cancer [1]. Regardless of pathological nodal status, research data indicated a considerable oncological advantage in PLND cohorts than non-PLND cohorts [15]. Along with the benefits of PLND, it also brings related complications. Symptomatic pelvic lymphocele, development of lymphoedema, ileus, deep venous thrombosis are the most common postoperative complications of PLND [23]. Since RC could already bring many complications, it might be difficult for elderly individuals to undergo RC with PLND [24, 25].

According to the Bladder Cancer Collaborative, the average number of LNs removed for bladder cancer should exceed 12.5 [26]. The average number of dissected LNs among all patients at our center was 10.46, which was less than the recommended. The main reason was that some elderly patients didn’t receive PLND. The average age of the patients who didn't receive PLND was higher than that of dissected patients (75.0 vs. 64.3, *P* < 0.001).

In some reports, the extended and super-extended PLND could bring a superior OS than standard PLND [15]. In a prospective phase III RCT, however, extended PLND failed to demonstrate better benefit to standard PLND [27]. It remains unclear whether the number of removed LNs is the most critical prognosticator. Besides, Herr et al. [26] reported that a minimum of 10 to 14 LNs should be dissected in RC. Thus, we chose patients who had 10 to 15 LNs removed and met all the other four criteria of Pentafecta as the subgroup. The subgroup (10–15 LNs) had a similar OS to the Pentafecta attained group (≥ 16 LNs). This result suggested that it might be unnecessary to remove as many as LNs possible, dissecting fewer LNs (10 LNs) could also bring the same prognostic benefits to patients.

In our center, PLND was associated with a favorable OS for MIBC patients (Fig. 4D). Interestingly, our findings indicated that PLND execution failed to improve the OS of NMIBC (Fig. 4C), whereas no PLND had a greater benefit in lowering complications in patients with negative LNs. With large sample sizes, a significant positive correlation between age and the perioperative complication rate was observed in RC for bladder cancer [4, 28]. In the real-world, high-risk NMIBC patients who received BCG instillations [29] or MIBC patients who received trimodality therapy [30] did not have any lymph node dissection. For patients who are capable of receiving BCG instillations but choose on RC, PLND will result in additional complications. Thus, whether to perform PLND or not in these patients, especially those with high-risk NMIBC, was still needed to be validated in future. This could also be the reason leading to low ≥ 16 LNs removed rate (30.3%) in our center.

According to the American National Cancer Database, 12.7% of NMIBC patients who underwent RC between 2004 and 2013 had positive LNs [31]. Remarkably, because they used the clinical stage, they misdiagnosed a portion of MIBC patients as NMIBC, resulting in a high positive rate. However, even in NMIBC patients at our center, positive LNs are also detected in 3.2% of patients (Additional file 2: Table S1). Thus, even in patients with NMIBC, the careful evaluation of the risk of LN metastases prior to surgery was still necessary, especially when PLDN was not intended to perform. In summary, Pentafecta has the potential to be a useful tool for evaluating the quality of radical cystectomy. 10 LNs removed may be more beneficial for bladder cancer patients requiring RC and PLND.

## Conclusions

Pentafecta is suitable for bladder cancer patients receiving LRC and has the potential to be a useful tool for evaluating the quality of LRC. Based on Pentafecta analysis, removing 10 LNs instead of 16 LNs as the one of the five criteria may be more appropriate for bladder cancer patients.

## Supplementary Information

**Additional file 1**. Raw data.

**Additional file 2**. Supplementary figure and table.

## Data Availability

All data generated or analyzed during this study are included in supplementary information files.

## Abbreviations

MIBC: Muscle invasive Bladder Cancer
NMIBC: Non-muscle invasive Bladder Cancer
RC: Radical cystectomy
PLND: Pelvic lymph node dissection
LN: Lymph node
OS: Overall survival
CI: Confidence interval
TMT: Trimodality therapy
BCG: Bacillus Calmette-Guerin
