# Supplementary material for: External validation of Pentafecta in patients undergoing laparoscopic radical cystectomy: results from a high-volume center
Source: BMC Urol. 2022 Mar 21;22:41. doi: 10.1186/s12894-022-00987-9 (PMC8939065; doi:10.1186/s12894-022-00987-9)
Supplement: Supplementary file 2 — Additional file 2. Supplementary figure and table. [file 12894_2022_987_MOESM2_ESM.docx]

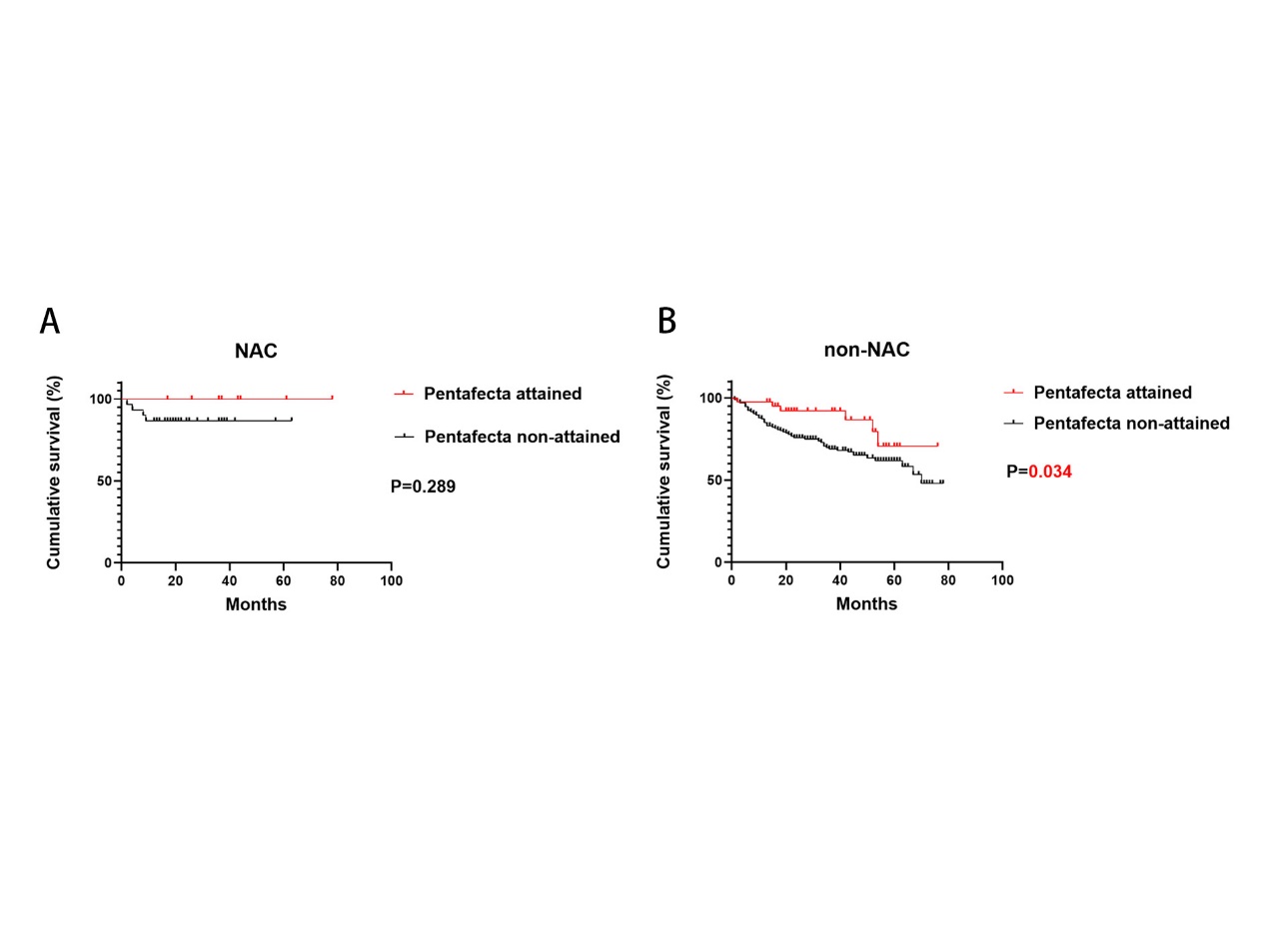


Figure S1. Subgroup survival analysis on NAC

A. In NAC patients, compare the Pentafecta attained group with Pentafecta non-attained group in OS.

B. In non-NAC patients, compare the Pentafecta attained group with Pentafecta non-attained group in OS.

Table S1. Lymph node positive rate in NMIBC and MIBC patients

|  | NMIBC | | MIBC | |
| --- | --- | --- | --- | --- |
|  | no PLND (n, %) | PLND (n, %) | no PLND (n, %) | PLND (n, %) |
| LN positivity |  |  |  |  |
| Negative nodes | 0 | 120 (96.8) | 0 | 129 (86.6) |
| Positive nodes | 0 | 4 (3.2) | 0 | 20 (13.4) |
| Unknown | 26 (100) | 0 | 41 (100) | 0 |
